# Sex-related differences in oncological surgery and postoperative outcomes: comprehensive, nationwide study in France

**DOI:** 10.1093/bjs/znae179

**Published:** 2024-08-16

**Authors:** Floriane Jochum, Anne-Sophie Hamy, Paul Gougis, Élise Dumas, Beatriz Grandal, Mathilde Sauzey, Enora Laas, Jean-Guillaume Feron, Virginie Fourchotte, Thomas Gaillard, Noemie Girard, Lea Pauly, Elodie Gauroy, Lauren Darrigues, Judicael Hotton, Lise Lecointre, Fabien Reyal, Fabrice Lecuru, Cherif Akladios

**Affiliations:** Residual Tumour and Response to Treatment Laboratory, RT2Lab, Translational Research Department, INSERM, U932 Immunity and Cancer, Paris, France; Department of Gynaecology, Strasbourg University Hospital, Strasbourg, France; Residual Tumour and Response to Treatment Laboratory, RT2Lab, Translational Research Department, INSERM, U932 Immunity and Cancer, Paris, France; Department of Medical Oncology, Institut Curie, Université Paris Cité, Paris, France; Residual Tumour and Response to Treatment Laboratory, RT2Lab, Translational Research Department, INSERM, U932 Immunity and Cancer, Paris, France; Residual Tumour and Response to Treatment Laboratory, RT2Lab, Translational Research Department, INSERM, U932 Immunity and Cancer, Paris, France; Residual Tumour and Response to Treatment Laboratory, RT2Lab, Translational Research Department, INSERM, U932 Immunity and Cancer, Paris, France; Department of Breast and Gynaecological Surgery, Institut Curie, Paris, Université Paris Cité, Paris, France; Residual Tumour and Response to Treatment Laboratory, RT2Lab, Translational Research Department, INSERM, U932 Immunity and Cancer, Paris, France; Department of Breast and Gynaecological Surgery, Institut Curie, Paris, Université Paris Cité, Paris, France; Department of Breast and Gynaecological Surgery, Institut Curie, Paris, Université Paris Cité, Paris, France; Department of Breast and Gynaecological Surgery, Institut Curie, Paris, Université Paris Cité, Paris, France; Department of Breast and Gynaecological Surgery, Institut Curie, Paris, Université Paris Cité, Paris, France; Department of Breast and Gynaecological Surgery, Institut Curie, Paris, Université Paris Cité, Paris, France; Department of Breast and Gynaecological Surgery, Institut Curie, Paris, Université Paris Cité, Paris, France; Department of Breast and Gynaecological Surgery, Institut Curie, Paris, Université Paris Cité, Paris, France; Department of Breast and Gynaecological Surgery, Institut Curie, Paris, Université Paris Cité, Paris, France; Department of Breast and Gynaecological Surgery, Institut Curie, Paris, Université Paris Cité, Paris, France; Department of Surgical Oncology, Institut Godinot, Reims, France; Department of Gynaecology, Strasbourg University Hospital, Strasbourg, France; Residual Tumour and Response to Treatment Laboratory, RT2Lab, Translational Research Department, INSERM, U932 Immunity and Cancer, Paris, France; Department of Breast and Gynaecological Surgery, Institut Curie, Paris, Université Paris Cité, Paris, France; Department of Breast and Gynaecological Surgery, Institut Curie, Paris, Université Paris Cité, Paris, France; Department of Gynaecology, Strasbourg University Hospital, Strasbourg, France

## Abstract

**Background:**

The main objective of this study was to undertake an exhaustive investigation of sex-related differences in cancer surgery.

**Methods:**

This observational study used data from the French national health insurance system database covering 98.8% of the population. Patients diagnosed with non-sex-specific solid invasive cancers between January 2018 and December 2019 were included. The main outcomes were likelihood of undergoing cancer surgery, type of oncological surgery performed, and associated 30-, 60-, and 90-day postoperative reoperation and mortality rates, by sex.

**Results:**

For the 367 887 patients included, women were 44% more likely than men to undergo cancer surgery (OR 1.44, 95% c.i. 1.31 to 1.59; *P* < 0.001). However, the likelihood of surgery decreased with advancing age (OR 0.98, 0.98 to 0.98; *P* < 0.001), and with increasing number of co-morbid conditions (OR 0.95, 0.95 to 0.96; *P* < 0.001), especially in women. Men had higher 90-day reoperation (21.2 *versus* 18.8%; *P* < 0.001) and mortality (1.2 *versus* 0.9%; *P* < 0.001) rates than women, overall, and for most cancer types, with the exception of bladder cancer, for which the 90-day mortality rate was higher among women (1.8 *versus* 1.4%; *P* < 0.001). After adjustment for age, number of co-morbid conditions, and surgical procedure, 90-day mortality remained higher in men (OR 1.16, 1.07 to 1.26; *P* < 0.001), and men were 21% more likely than women to undergo reoperation within 90 days (OR 1.21, 1.18 to 1.23; *P* < 0.001).

**Conclusion:**

Women were much more likely than men to undergo cancer surgery than men, but the likelihood of surgery decreased with advancing age and with increasing number of co-morbid conditions, especially in women. These findings highlight a need for both increased awareness and strategies to ensure gender equality in access to oncological surgical treatment and improved outcomes.

## Teaser

This study comprised a comprehensive analysis of sex- and gender-related differences in oncological surgical procedures using data from the French national health insurance system. It found that women were more likely to undergo cancer surgery than men, but faced a decreased likelihood with age and co-morbidities. Men had higher rates of reoperation and mortality after surgery for the majority of cancer types. The results call for enhanced efforts to achieve gender equality in cancer surgery outcomes.

## Introduction

Sex and gender are two distinct concepts in healthcare. Sex is based on biological factors, whereas gender is tied to societal roles, behaviours, and life experiences^1^. Gender differences play a crucial role in shaping healthcare experiences, including access to care, healthcare system use, and the attitudes of medical staff^2^. Prevailing gender differences in healthcare encapsulate disparities in access to preventive medicine, drug-prescribing patterns, and referral for, or acceptance of, surgical treatments.

In oncology, there is evidence to suggest that men and women may not always receive identical treatments, even when clinically recommended. For example, women diagnosed with head-and-neck^3^, bladder^4^, gastro-oesophageal^5,6^ or colorectal cancers^7^ are less likely to undergo intensive chemoradiotherapy than men. These disparities in chemotherapy use have been attributed to differences between the sexes in tumour characteristics, such as histology and stage, but also to gender-related factors, such as an increased time to diagnosis^4^. Treatment response may also differ between men and women^8^. In a recent study^9^ evaluating adverse events in 23 296 patients from 202 trials, women were more likely than men to experience severe adverse effects from cancer treatments such as chemotherapy, targeted therapy, and immunotherapy. This disparity was most pronounced among patients receiving immunotherapy, with women having a risk of serious adverse effects almost 50% higher than that of men. Sex-related elements, such as differences in average body type or pharmacokinetics, have been identified as possible causes of these disparities, but gender-related differences in symptom perception could also play a significant role.

In the field of oncological surgery, limited data are available concerning sex- and gender-related differences. Women with lung cancer are more likely to be diagnosed at earlier stages than men with the same disease^10^ and to undergo surgery for locoregional disease^11^, whereas the opposite pattern has been reported for bladder cancer^12^. In colorectal cancer, men are more frequently diagnosed with left colonic and rectal cancer^13^, and have higher rates of postoperative complications than women^14^. However, even though surgical intervention is often the primary treatment for invasive solid cancers, no comprehensive and extensive analysis of sex- and gender-related differences in surgical treatment and/or associated reoperation rates has ever been published.

This study aimed to assess the interaction between sex and the likelihood of receiving cancer surgery, the type of oncological surgery performed, and the associated 30-, 60-, and 90-day reoperation and mortality rates using an extensive nationwide database.

## Methods

### Patient data and selection

Data were obtained from the French national health insurance system database, which covers 98.8% of the 67 million inhabitants of France. All medical and administrative information relating to the reimbursement of French citizens for hospital healthcare expenses is collected and aggregated within this system. Patients diagnosed with non-sex-specific invasive solid cancers (excluding ovarian, uterine, cervical, vulvar, vaginal, penile, testicular, and prostate cancers) between January 2018 and December 2019 were selected (*Supplementary material*). Patients with an existing cancer diagnosis in 2016 and 2017 were excluded from the study to ensure the identification of incident cases only. Patients under 18 years of age and those with gender coding discrepancies were also excluded. Discrepancies were identified as data recording errors, not indicative of patients undergoing sex change. Finally, skin cancers other than melanoma, breast cancer, and patients with several concomitant cancers were excluded from the study. This study followed the STROBE and SAGER guidelines^15^ (*supplementary material*), and was authorized by the French data protection agency (registration number 2204867v0).

### Definition of patient’s sex

The French national health insurance system database traditionally records sex as a binary attribute (male/female), reflecting biological distinctions established at birth and reflected on identification documents. Non-binary and LGBTQ+ (lesbian, gay, bisexual, transgender, queer, and others) populations could not be assessed within the scope of this study.

### Clinical and surgical characteristics

The clinical features assessed in this study included sex, age, co-morbid conditions, and type of cancer. Age at diagnosis was analysed as a continuous variable. Co-morbid conditions were identified in the database from the main, relative, and associated diagnoses made during hospital stays, and medical and surgical procedures undergone during hospital stays (*supplementary material*). All clinical characteristics were described for the overall population and by sex.

Cancer surgery and associated endpoints were analysed specifically for the 13 most prevalent types of cancer identified in the cohort, including colorectal, lung, bladder, melanoma, kidney, pancreas, liver and biliary tract, thyroid, central nervous system, stomach, pharynx, oesophagus, and larynx. Anal cancer was excluded from the colorectal cancer category in all analyses related to cancer surgery. Only surgical acts corresponding to staging or curative cancer surgery were selected.

### Endpoints

Endpoints included the likelihood of undergoing cancer surgery, the specific type of oncological procedure performed, and the associated 30-, 60-, and 90-day postoperative reoperation and mortality rates (*supplementary material*); these endpoints were compared by sex.

### Statistical analysis

The study population is described in terms of frequencies for qualitative variables, and median (i.q.r.) for quantitative variables. Comparisons were performed using χ² tests for qualitative variables, and Mann–Whitney or Kruskal–Wallis tests for quantitative variables. Additionally, for assessing the effect size for differences in mortality and reoperation rates between groups, Cohen's h was calculated, providing a standardized measure of the magnitude of these differences.

Factors associated with the likelihood of undergoing cancer surgery were investigated using a mixed multivariable logistic regression model, with cancer type as a random effect. Sex, age, and number of co-morbidities were included as fixed-effect variables. Interactions between these variables and patient sex were checked for. Similarly, a multivariable logistic regression model was used to assess the likelihood of 90-day postoperative reoperation and mortality. Sex, age, number of co-morbidities, and type of oncological procedure performed were included in these models. There were no missing data. All statistical analyses were done using R 4.0.3 (R Foundation for Statistical Computing, Vienna, Austria).

## Results

### Patient characteristics

**Fig. 1:**
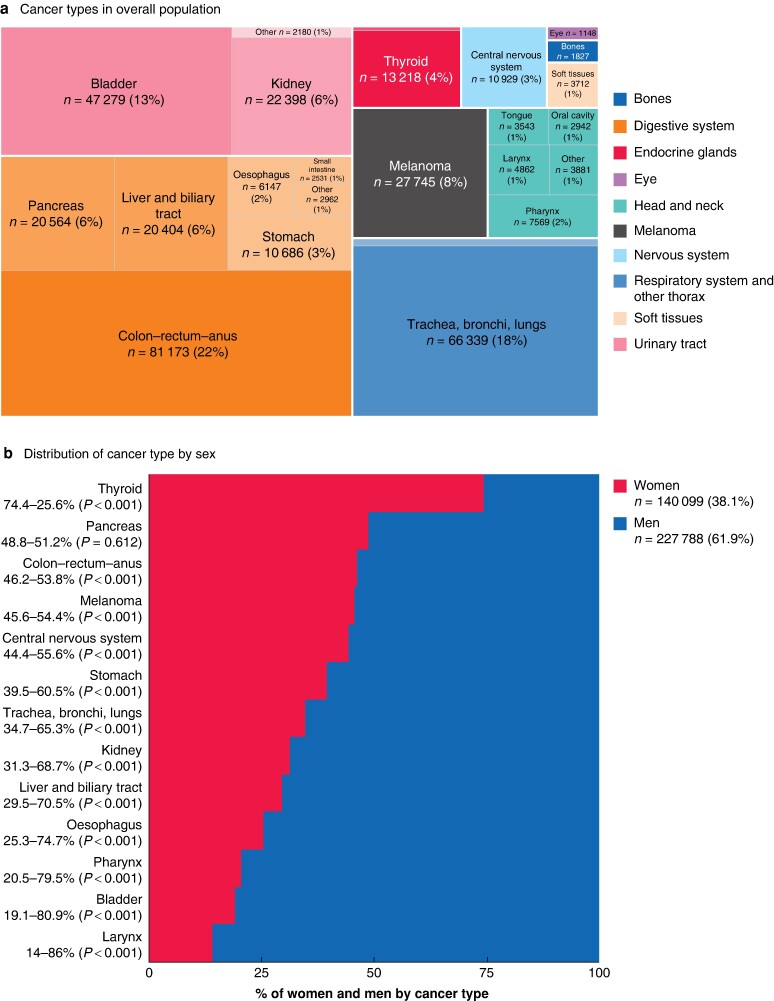

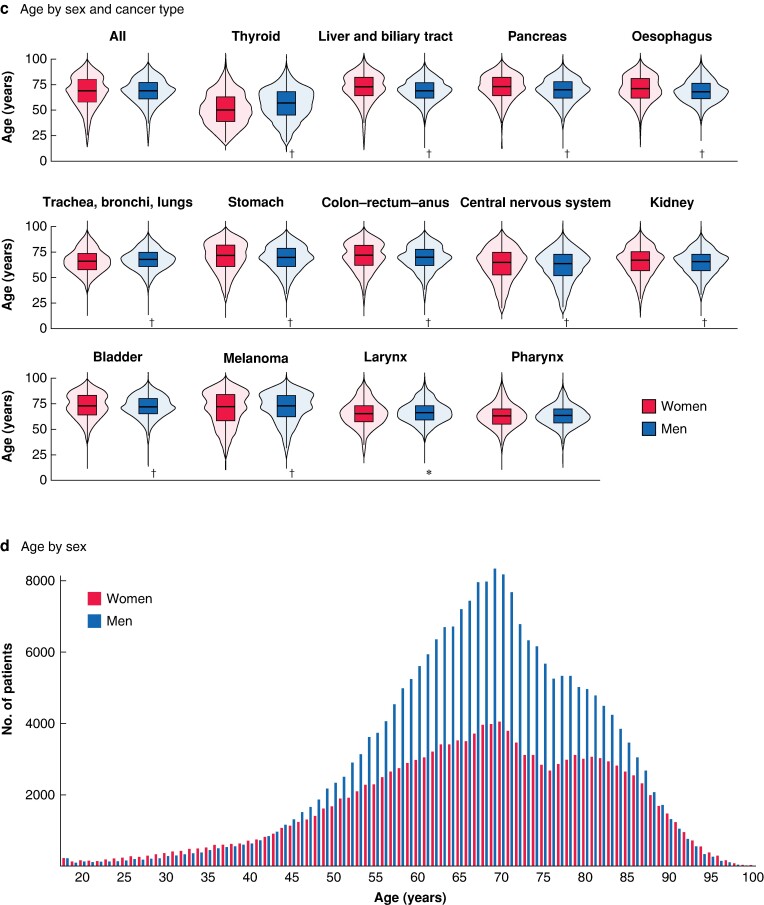
Distribution of cancer types and age by sex **a** Distribution of cancer types in overall population. **b** Percentage distribution of cancer types by sex. **c** Age distribution by sex and cancer type. Horizontal lines, boxes, and outer shaded areas represent the median, interquartile range, and kernel density estimate of the age distribution respectively. *P < 0.050, †*P* < 0.001 *versus* women (Mann-Whitney test). **d** Age distribution by sex.

In total, 367 887 patients were included (140 099 women, 38.1%; 227 788 men, 61.9%). The most frequently diagnosed cancer overall was colorectal cancer (81 173 patients) followed by lung and bladder cancer (*Fig. 1a*,*b*). The type of cancer varied by sex; thyroid and pancreatic cancer were more common in women, whereas all other cancer types examined were significantly more frequent in men (*Fig. 1c*).

Median age at cancer diagnosis was 69 (i.q.r. 60–78) years overall, 69 (58–80) years for women, and 69 (61–77) years for men (*P* = 0.094) (*Fig. 1d*). Median age- and sex-related differences were statistically significant in thyroid cancer (50 years in women *versus* 57 years in men; *P* < 0.001) and liver and biliary tract cancers (73 years in women *versus* 69 years in men; *P* < 0.001).

### Co-morbid conditions

**Fig. 2:**
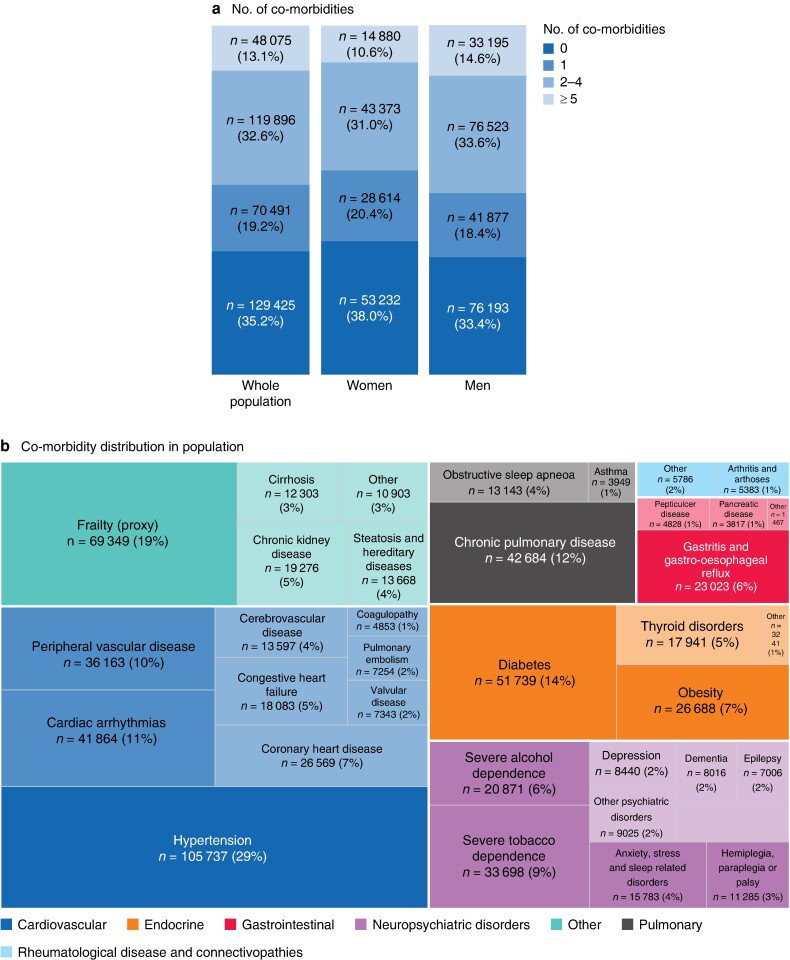

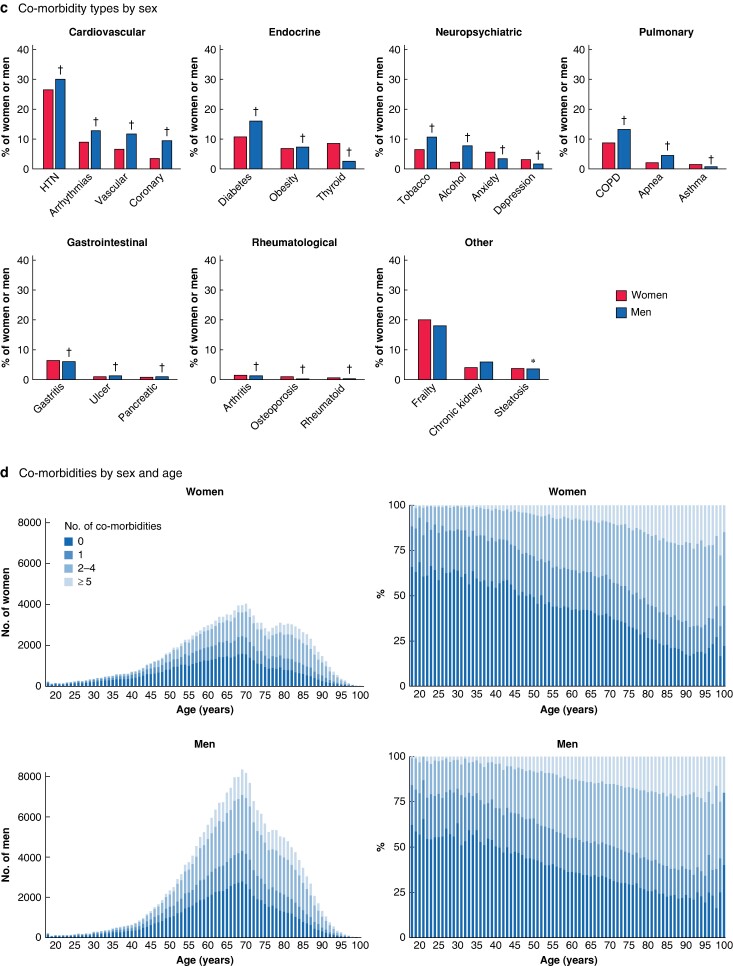
Distribution of co-morbidities **a** Number of co-morbidities in overall population and by sex. **b** Distribution of co-morbidity types in the overall population. **c** Distribution of co-morbidity types by sex. HTN, hypertension; COPD, chronic obstructive pulmonary disease. *P < 0.050, †*P* ≤ 0.001 *versus* women (χ² test). **d** Number of co-morbidities by age and sex.

Overall, at the time of cancer diagnosis, 62.0% of women and 66.6% of men had at least one co-morbid condition (*P* < 0.001) (*Fig. 2a*). The three most common co-morbid conditions were hypertension (28.7%), diabetes (14.1%), and chronic pulmonary disease (11.6%); a frailty pattern was present in 18.9% of the patients (*Fig. 2b*). The prevalence of co-morbid conditions varied significantly between the sexes (*Fig. 2c*), with men generally having a higher incidence than women, except for rheumatological diseases, anxiety, depression, asthma, and thyroid pathologies. The frequencies and numbers of co-morbid conditions were markedly higher in men than in women across all age groups until 80 years of age (*Fig. 2d*).

### Predicted likelihood of undergoing cancer surgery

**Fig. 3:**
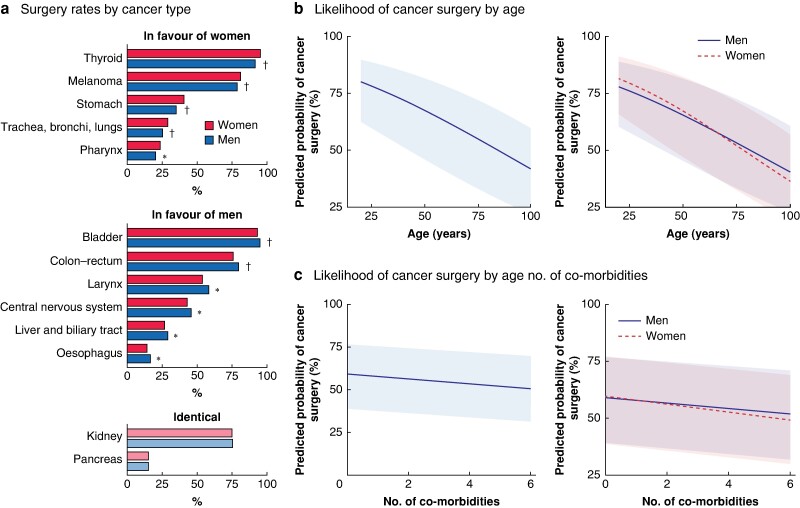
Likelihood of receiving cancer surgery **a** Percentage of women and men who underwent cancer surgery by cancer type. *P < 0.050, †*P* < 0.001 *versus* women (χ² test). **b** Age-based results from logistic multivariable analyses predicting the likelihood of receiving cancer surgery overall (*P <* 0.001) and by sex (*P <* 0.001 for interaction between women and men). **c** Results pertaining to the number of co-morbidities from logistic multivariable analyses predicting the likelihood of receiving cancer surgery overall (*P <* 0.001) and by sex (*P <* 0.001 for interaction between women and men).

Overall, for the most prevalent cancer types, 69 259 women (58.2%) and 115 251 men (57.6%) underwent cancer surgery (*P* < 0.001) (*Fig. 3a*). Surgery rates were highest for thyroid cancer (94.4%), and lowest for pancreatic cancer (15.0%). Women underwent surgery statistically significantly more often than men for thyroid, melanoma, stomach, lung, and pharyngeal cancer. Men had statistically significantly higher surgery rates than women for bladder, colorectal, larynx, central nervous system, liver and biliary tract, and oesophageal cancers. No sex-related differences in surgery rates were observed for kidney and pancreatic cancers.

After accounting for age, number of co-morbidities, and cancer type, the adjusted multivariable analysis showed that women had a 44% higher likelihood of undergoing cancer surgery than men (OR 1.44, 95% c.i. 1.31 to 1.59; *P* < 0.001). The predicted probability of cancer surgery decreased with increasing age (OR 0.98, 0.98 to 0.98; *P* < 0.001), and number of co-morbidities (OR 0.95, 0.95 to 0.96; *P* < 0.001). Significant interactions between sex, age, and number of co-morbidities underscore the complex interplay these factors have on surgical decisions, revealing that the increased likelihood of surgery for women compared with men is influenced negatively by older age and a higher number of co-morbidities (*P* < 0.001 for both interactions (*Fig. 3b*,*c* and  *Table S1*).

### Type of oncological surgery by sex

Analysis of oncological surgical procedures first revealed numerous sex-related variations in relation to cancer epidemiology (*Fig. S1*). Right colectomy was performed more frequently in women for colorectal cancer (26.8% *versus* 21.1% for men; *P* < 0.001), whereas men were more likely to undergo rectal resection (20.5% *versus* 14.7% for women; *P* < 0.001) and left colectomy (17.1 *versus* 14.2%; *P* < 0.001). Similarly, cholecystectomy was more frequently performed in women with liver and biliary tract cancers (10.8% *versus* 6.8% for men; *P* < 0.001), and men were more likely to undergo hepatic resection (24.4 *versus* 17.9%; *P* < 0.001). For central nervous system cancers, brain tissue removal was undertaken more frequently in men than in women (40.0 *versus* 35.8%; *P* < 0.001), whereas women were more likely to undergo removal of extraencephalic intracranial tumours (2.6 *versus* 1.6%; *P* < 0.001).

**Fig. 4:**
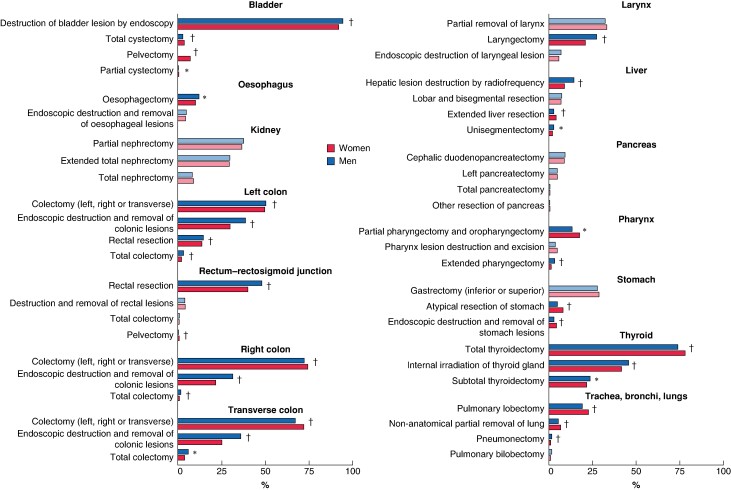
Surgical interventions by cancer type and sex for the most prevalent cancer types This analysis focused on cancer types for which the extent and aggressiveness of surgical intervention could be assessed, offering insights into variations in surgical treatment approaches beyond biological differences. Colorectal cancer was divided into left colon, right colon, transverse colon, and rectal–rectosigmoid junctional cancers, and liver cancer was separated from biliary tract cancer. Percentages by cancer type may exceed 100% owing to patients undergoing multiple surgical procedures. *P < 0.050, †*P* ≤ 0.001 *versus* women (χ² test).

To minimize the impact of sex-specific cancer epidemiology and enhance the analysis of potential sociocultural gender differences, a secondary examination of oncological surgical procedures was conducted. This analysis focused on cancer types for which the extent and aggressiveness of surgical intervention could be assessed, offering insights into variations in surgical treatment approaches beyond biological differences (*Fig. 4*). For this analysis, colorectal cancer was divided into left colonic, right colonic, transverse colonic, and rectal–rectosigmoid junctional cancers, and liver cancer was separated from biliary tract cancer. For conditions such as bladder, colonic and liver cancer, women tended to undergo more aggressive or extensive procedures than men. Specifically, 11.1% of women with bladder cancer received total cystectomy/pelvectomy, compared with only 2.9% of men (*P* < 0.001). Similarly, women with colonic cancer were more frequently subjected to colectomy, whereas men were inclined towards less invasive endoscopic removals of lesions. Women with liver cancer received more extended liver resections than men (4.3 *versus* 2.9%; *P* < 0.001). Conversely, the surgical treatment for lung, pharyngeal, and stomach cancers showed a different trend, with men receiving more extensive procedures. Men with lung cancer were more likely to undergo pneumonectomy (1.8 *versus* 1.1%; *P* < 0.001), whereas women were more frequently treated by pulmonary lobectomy (22.7 *versus* 19.3%; *P* < 0.001) or non-anatomical partial lung removal (6.8 *versus* 5.5%; *P* < 0.001). Pharyngeal cancer treatments also differed, with women being more likely to undergo partial laryngectomy, whereas full laryngectomies were performed predominantly in men. Similarly, for stomach cancer, women were more often subjected to endoscopic lesion removal than men.

### Postoperative reoperation and mortality by sex

**Fig. 5:**
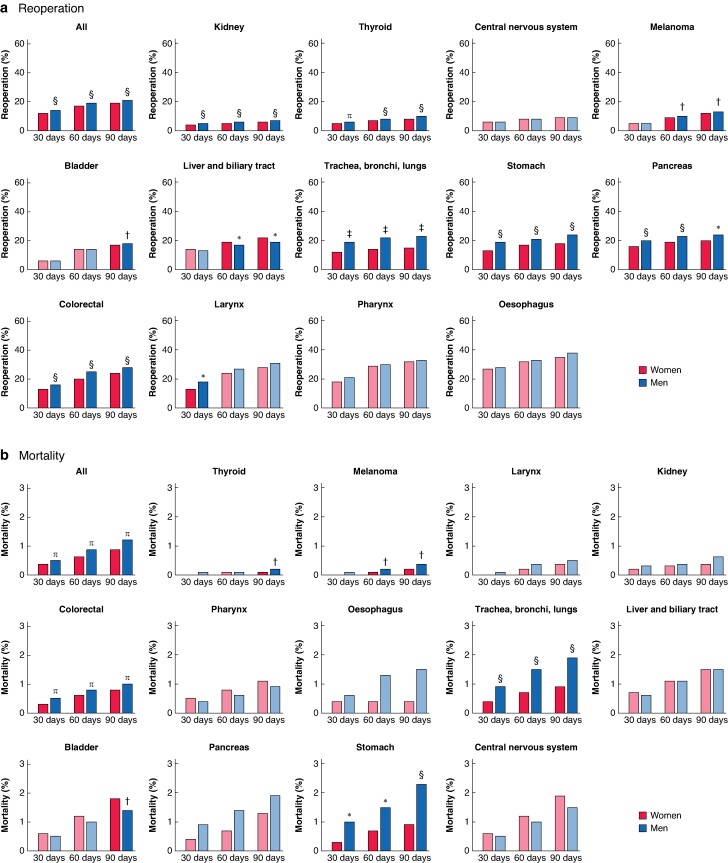
Distribution of 30-, 60-, and 90-day postoperative reoperation and mortality rates by cancer type and sex **a** Reoperation rate and **b** mortality rate. Cohen’s h values have been included to quantify the effect size, enhancing the interpretability of these differences. A Cohen’s h value lower than 0.1 indicates a very small effect size. *P < 0.050 (h = 0.1), †*P* = 0.050 (h < 0.1), ‡*P* < 0.001 (h = 0.2), §*P* < 0.001 (h = 0.1), ¶*P* < 0.001 (h < 0.1) *versus* women (χ² test).

The overall rate of reoperation within 90 days was 20.3% (*Fig. 5a*). The highest rate of postoperative reoperation was for oesophageal cancer, for which 37.2% of patients had undergone reoperation within 90 days. The 90-day reoperation rate was significantly higher for men than for women for all cancer types (21.2 *versus* 18.8%; *P* < 0.001), and remained statistically higher for kidney, thyroid, melanoma, bladder, lung, stomach, pancreatic, and colorectal cancers. The 90-day reoperation rate was significantly higher for women than men for liver and biliary tract cancers. After adjustment for age, number of co-morbid conditions, and surgical procedure performed, men remained at higher risk of reoperation within 90 days and were 21% more likely than women to undergo reoperation (OR 1.21, 95% c.i. 1.18 to 1.23; *P* < 0.001) (*Fig. S2*).

The 90-day mortality rate after cancer surgery was 1.1% (*Fig. 5b*). Mortality was highest for central nervous system cancer, with a 90-day mortality rate of 1.7%. The overall 90-day mortality rate was significantly higher for men than for women (1.2% *versus* 0.9%; *P* < 0.001), and this statistically significant difference remained for thyroid, melanoma, colorectal, lung, and stomach cancers. In bladder cancer, the 90-day mortality rate was statistically significantly higher for women than men. After adjustment for age, number of co-morbid conditions, cancer type, and surgical procedure performed, men remained at higher risk of death within 90 days of surgery (OR 1.16, 1.07 to 1.26; *P* < 0.001) (*Fig. S3*).

## Discussion

In this large-scale study involving 367 887 patients, women were 44% more likely than men to undergo cancer surgery, but women were less likely than men to undergo surgery if they had multiple co-morbid conditions or were elderly. There were significant differences in the prevalence of co-morbid conditions between the sexes at the time of cancer diagnosis, with men being 7% more likely to have at least one co-morbidity than women. The higher burden of co-morbid conditions in men observed here is consistent with the known higher rates of risk factors associated with male sex, such as smoking and excessive alcohol consumption^16^. This finding echoes the authors’ previous work, in which elderly women and women with co-morbid conditions were found to be more likely to be referred to non-expert cancer facilities, highlighting the importance of considering sociocultural gender-specific differences in clinical decision-making^17^.

The present investigation into cancer surgery also highlighted sex-dependent differences in the types of oncological surgery performed. Women were more likely to present with right-sided colonic cancer and consequently to undergo right colectomy, whereas men more frequently underwent left colectomy or rectal resection for colorectal cancer. Both genetic and environmental factors (for example dietary factors) are believed to play roles in sex-related differences in the tendency to have right- or left-sided colonic cancers^13^. Although the present findings indicate significant sex-related differences in colonic cancer procedures, the literature on this subject has been mixed, with some studies^18,19^ reporting that women are more likely to undergo surgery, but others^20^ documenting only relatively small differences in treatment allocation between the sexes. Similar sex-specific patterns were observed here in terms of surgery for liver and biliary tract cancers, and those of the central nervous system. Surgeons’ awareness of these sex-specific disease locations is crucial for the timely recognition of symptoms, minimization of diagnostic delays, and ensuring preparedness for unique surgical challenges.

In addition to such sex-related differences in types of oncological surgery performed, this study has also highlighted the sociocultural gender-related disparities in types of oncological procedure undertaken. Notably, it was found that women are more likely to receive aggressive or extensive surgical interventions than men for bladder, colonic, and liver cancers. Even though bladder cancer is diagnosed four times more frequently in men than in women, adverse survival outcomes are more likely in women^21^. Women with bladder cancer typically present at more advanced clinical stages, largely owing to delays in the evaluation of haematuria. A prospective cohort study^22^ noted that, among patients with clinical symptoms, 84.0% of men and 66.7% of women consulted a urologist directly. One-third of the women saw a general practitioner and/or gynaecologist once or twice before referral to the urologist, and women were significantly more frequently treated for urinary tract infections than men (61.1 *versus* 20.0%; *P* ≤ 0.010). Consequently, women more frequently underwent total cystectomy in the present study, whereas men were predominantly treated by endoscopic resection. However, it is important to consider that some studies attributed these disparities to biological factors inherent to sex affecting the muscle-invasiveness of bladder cancer, such as hormonal influences and genetic predispositions, rather than sociocultural influences, suggesting a complex interplay of both elements^23^. Conversely, women with lung cancer were diagnosed earlier than men with the same disease, and benefited from more localized and less invasive surgical interventions. A previous study^10^ showed that men were consistently more likely than women to be diagnosed at stage III–IV, for all lung cancer types, cancer registries, smoking behaviours, and socioeconomic backgrounds. In our study, a similar conclusion was drawn for stomach and pharyngeal cancers, with women undergoing surgery more frequently than men. These differences highlight the need for improvements in prevention and diagnostic evaluations for lung, stomach, and pharyngeal cancers in men, and for bladder, colonic and liver cancers in women.

There are limitations to this study. The major limitation is the absence of data on disease stage and histological type, which are crucial variables for comprehensively understanding the observed differences. This limitation prevents assessment of whether disparities are related to biological sex-related factors or to gender-related factors, such as differential access to care or treatment biases. Additionally, the binary gender classification in the French national health insurance system database does not encompass the full spectrum of gender identities, potentially oversimplifying the impact of gender on oncological and surgical outcomes. Finally, the analysis of 90-day reoperation rate does not differentiate between planned and unplanned procedures, which could skew understanding of postoperative outcomes, particularly where a subsequent operation comprises a scheduled part of the treatment process and a sign of positive recovery. Despite these limitations, to the authors’ knowledge, this is the first study to provide a comprehensive overview of oncological surgery and reoperation rates by sex across multiple cancer types on a larger scale. The study has revealed consistently higher reoperation rates in men than women, and confirmed previous observations of higher mortality and complication rates in men, particularly for lung^24^ and gastric^25^ cancers, highlighting the importance of comprehensive assessment of co-morbid conditions and the optimization of perioperative care in male patients with cancer.

This study has highlighted the multifaceted nature of healthcare disparities in cancer, disentangling the influence of sex, gender, age, and co-morbid conditions on diagnosis, treatment, and outcomes. There is a need to develop comprehensive strategies that not only increase accessibility to healthcare, but also prioritize understanding and addressing the underlying causes of disparities in oncological surgery by: recognizing and mitigating the effects of sex and gender biases in clinical decision-making; incorporating comprehensive patient assessments, including evaluations of co-morbid conditions acknowledging potential sex-specific risks; improving preventive measures and diagnostic procedures; and developing and implementing guidelines for personalized treatment plans and postoperative protocols taking patient sex, age, and overall health condition into account. Future studies, including histological and staging data, should continue to investigate these sex- and gender-specific differences to guide tailored strategies for improving cancer care for all patients.

## Supplementary Material

znae179_Supplementary_Data

## Data Availability

The data that support the findings of this study are not publicly available owing to the conditions of access to the data of the French national health insurance system database. Interested individuals can apply to the Agence Technique de l'Information sur l'Hospitalisation (https://www.atih.sante.fr) or Health Data Hub (https://www.health-data-hub.fr), and once approved, can apply to the corresponding author.
