# Supplementary material for: Sex-related differences in oncological surgery and postoperative outcomes: comprehensive, nationwide study in France
Source: Br J Surg. 2024 Aug 16;111(8):znae179. doi: 10.1093/bjs/znae179 (PMC11327872; doi:10.1093/bjs/znae179)
Supplement: znae179_Supplementary_Data [file znae179_supplementary_data.docx]

**Sex-related Differences in Oncological Surgery and Postoperative Outcomes: A Comprehensive, Nationwide Study in France**

Floriane Jochum, MD^1;2^, Anne-Sophie Hamy, PhD^1;3^, Paul Gougis, MD^1^, Élise Dumas, PhD^1^, Beatriz Grandal, MD^1;4^, Mathilde Sauzey^1;4^, Enora Laas, MD^4^, Jean-Guillaume Feron, MD^4^, Virginie Fourchotte, MD^4^, Thomas Gaillard, MD^4^, Noemie Girard, MD^4^, Lea Pauly, MD^4^, Elodie Gauroy, MD^4^, Lauren Darrigues, MD^4^, Judicael Hotton, MD^5^, Lise Lecointre, PhD^2^, Fabien Reyal, PhD^1;4^, Fabrice Lecuru, PhD^4*^, Cherif Akladios, PhD^2*^

^1^ Residual Tumor & Response to Treatment Laboratory, RT2Lab, Translational Research Department, INSERM, U932 Immunity and Cancer, Paris, France

^2^ Department of Gynecology, Strasbourg University Hospital, Strasbourg, France

^3^ Department of Medical Oncology, Institut Curie, Université Paris Cité, Paris, France

^4^ Department of Breast and Gynecological Surgery, Institut Curie, Paris, Université Paris Cité, France

^5^ Department of Surgical Oncology, Institut Godinot, Reims, France.

*Equal contributions

**Corresponding author:**

Pr Fabien REYAL

Work address: RT2lab, Institut Curie, 26 rue d’Ulm, 75005 Paris, France

E-mail: fabien.reyal@gmail.com

Tel: + 33 1 44 32 40 87

**Supplementary Materials - Index**

| **Supplementary Methods** |  |
| --- | --- |
| Flow-chart for patient selection | *page 3* |
| STROBE statement | *page 4* |
| Details on the methodology of comorbidities | *page 6* |
| Details on the endpoints | *page 7* |
|  |  |
| **Supplementary Figures and Tables** |  |
| Supplementary Figure 1. Detailed Analysis of Oncological Surgeries Highlighting Sex-Specific Variations in Relation to Cancer Epidemiology. | *page 8* |
| Supplementary Table 1. Findings from the multivariable logistic regression analysis predicting the likelihood of receiving cancer surgery  Supplementary Figure 2. Findings from the multivariable logistic regression analysis predicting 90-day reoperation  Supplementary figure 3. Findings from the multivariable logistic regression analysis predicting 90-day mortality | *page 9*  *page 10*  *page 12* |
|  |  |
| **References** | *page 14* |
|  |  |

# Supplementary Methods

# Supplementary Method 1. Flow-chart for patient selection


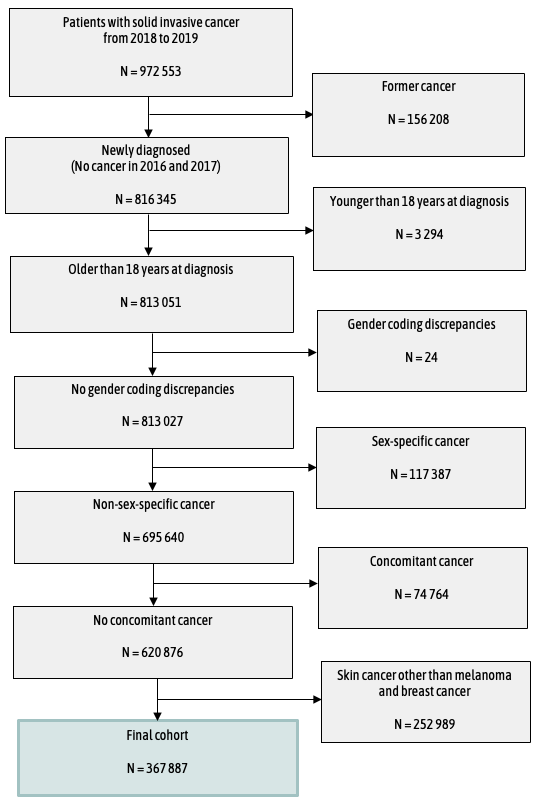


# Supplementary Method 2. STROBE Statement

STROBE Statement—Checklist of items that should be included in reports of ***cohort studies***

|  | Item No | Recommendation | Page No |  |
| --- | --- | --- | --- | --- |
| Title and abstract | 1 | (*a*) Indicate the study’s design with a commonly used term in the title or the abstract | Title page |  |
|  |  | (*b*) Provide in the abstract an informative and balanced summary of what was done and what was found | 2 |  |
| Introduction | | |  |  |
| Background/rationale | 2 | Explain the scientific background and rationale for the investigation being reported | 3 |  |
| Objectives | 3 | State specific objectives, including any prespecified hypotheses | 4 |  |
| Methods | | |  |  |
| Study design | 4 | Present key elements of study design early in the paper | 4 |  |
| Setting | 5 | Describe the setting, locations, and relevant dates, including periods of recruitment, exposure, follow-up, and data collection | 4 |  |
| Participants | 6 | (*a*) Give the eligibility criteria, and the sources and methods of selection of participants. Describe methods of follow-up | 4 |  |
|  |  | (*b*) For matched studies, give matching criteria and number of exposed and unexposed |  |  |
| Variables | 7 | Clearly define all outcomes, exposures, predictors, potential confounders, and effect modifiers. Give diagnostic criteria, if applicable | 5 |  |
| Data sources/ measurement | 8* | For each variable of interest, give sources of data and details of methods of assessment (measurement). Describe comparability of assessment methods if there is more than one group | 4-5 |  |
| Bias | 9 | Describe any efforts to address potential sources of bias | 5 |  |
| Study size | 10 | Explain how the study size was arrived at | 4 |  |
| Quantitative variables | 11 | Explain how quantitative variables were handled in the analyses. If applicable, describe which groupings were chosen and why | 4 |  |
| Statistical methods | 12 | (*a*) Describe all statistical methods, including those used to control for confounding | 6 |  |
|  |  | (*b*) Describe any methods used to examine subgroups and interactions | 6 |  |
|  |  | (*c*) Explain how missing data were addressed | 6 |  |
|  |  | (*d*) If applicable, explain how loss to follow-up was addressed |  |  |
|  |  | (*e*) Describe any sensitivity analyses | 6 |  |
| Results | | |  |  |
| Participants | 13* | (a) Report numbers of individuals at each stage of study—eg numbers potentially eligible, examined for eligibility, confirmed eligible, included in the study, completing follow-up, and analysed | 6-7 |  |
|  |  | (b) Give reasons for non-participation at each stage | 4 |  |
|  |  | (c) Consider use of a flow diagram | 4 |  |
| Descriptive data | 14* | (a) Give characteristics of study participants (eg demographic, clinical, social) and information on exposures and potential confounders | 6-7 |  |
|  |  | (b) Indicate number of participants with missing data for each variable of interest |  |  |
|  |  | (c) Summarise follow-up time (eg, average and total amount) |  |  |
| Outcome data | 15* | Report numbers of outcome events or summary measures over time | 7-10 |  |
| Main results | 16 | (*a*) Give unadjusted estimates and, if applicable, confounder-adjusted estimates and their precision (eg, 95% confidence interval). Make clear which confounders were adjusted for and why they were included | 7-10 |  |
|  |  | (*b*) Report category boundaries when continuous variables were categorized | 7-10 |  |
|  |  | (*c*) If relevant, consider translating estimates of relative risk into absolute risk for a meaningful time period |  |  |
| Other analyses | 17 | Report other analyses done—eg analyses of subgroups and interactions, and sensitivity analyses | 7-10 |  |
| Discussion | | |  |  |
| Key results | 18 | Summarise key results with reference to study objectives | 10-11 |  |
| Limitations | 19 | Discuss limitations of the study, taking into account sources of potential bias or imprecision. Discuss both direction and magnitude of any potential bias | 11-12 |  |
| Interpretation | 20 | Give a cautious overall interpretation of results considering objectives, limitations, multiplicity of analyses, results from similar studies, and other relevant evidence | 10-11 |  |
| Generalisability | 21 | Discuss the generalisability (external validity) of the study results | 12-13 |  |
| Other information | | |  |  |
| Funding | 22 | Give the source of funding and the role of the funders for the present study and, if applicable, for the original study on which the present article is based |  |  |

#

# Supplementary Method 3. Details on the methodology of comorbidities

We relied on previously published articles^1–10^ to define the list of considered comorbidities, together with the medical codes used to identify them in the French national health insurance database. Diagnosis codes were recorded with the International Classification of Diseases—10th revision, ICD-10^11^. Procedures were recorded with the CCAM classification (“Classification Communes des Actes Médicaux”). The final list of comorbidities included 51 pathologies, gathered into 12 categories: (1) Cardiovascular, (2) Endocrine, (3) Frailty, (4) Gastrointestinal, (5) Immune, (6) Kidney, (7) Liver, (8) Neurologic, (9) Psychiatric disorders, (10) Pulmonary, (11) Rheumatologic disease and connectivopathies, and (12) Other. A patient was suspected to suffer from a given comorbidity at the time of cancer diagnosis if there was at least one CCAM procedure code or ICD-10 diagnosis code associated with the given comorbidity in the year preceding the date of cancer diagnosis.

# Supplementary Method 4. Details on the endpoints

These endpoints were assessed for the fourteen most frequent cancer types of the cohort.

1. Likelihood of Undergoing Cancer Surgery: This endpoint reflects the likelihood of patients receiving surgical intervention following a diagnosis of cancer. It is calculated as the proportion of diagnosed cancer patients who underwent cancer surgery during the study period. Only the surgical acts corresponding to staging or curative cancer surgeries were selected.

2. Type of Oncological Procedure Performed: This endpoint identifies the specific surgical procedures employed in the treatment of cancer patients. The procedures were categorized based on the type of cancer diagnosed. In our initial analysis, which focused on sex-specific variations, we aggregated colon, rectum, and anus cancers, as well as liver and biliary tract cancers, into single categories. For the subsequent analysis, which delves into gender disparities, we refined our approach by further segmenting these categories. Only the surgical acts (CCAM code) corresponding to staging or curative cancer surgeries were selected. We used the official CCAM classification system to label each surgical act.

3. 30-Day Postoperative Reoperation Rate: This endpoint records the percentage of patients who had to undergo a second surgical procedure within 30 days following the initial oncological surgery. All instances of reoperation were included in our analysis, encompassing cases of reoperation due to complications, restaging procedures, or further diagnostic procedures.

4. 60-Day Postoperative Reoperation Rate: Similar to the 30-day rate, this endpoint records the percentage of patients who had to undergo a second surgical procedure within 60 days of the initial oncological surgery.

5. 90-Day Postoperative Reoperation Rate: Similar to the 30-day rate, this endpoint records the percentage of patients who had to undergo a second surgical procedure within 90 days of the initial oncological surgery.

6. 30-Day Postoperative Mortality Rate: This endpoint records the percentage of patients who died within 30 days of the oncological surgical intervention, providing an early measure of surgical mortality. Deaths were identified within the database using a hospital discharge code of 9.

7. 60-Day Postoperative Mortality Rate: This endpoint measures the percentage of patients who died within 60 days of the oncological surgical procedure.

8. 90-Day Postoperative Mortality Rate: This endpoint records the percentage of patients who died within 90 days of the oncological surgical intervention.

In each case, comparisons were made based on the sex of the patients, shedding light on potential disparities in these endpoints due to sex and gender-related factors.

# Supplementary Figures and Tables

#

# Supplementary Figure 1. Detailed Analysis of Oncological Surgeries Highlighting Sex-Specific Variations in Relation to Cancer Epidemiology. (A) This section provides a breakdown of the types of oncological surgeries performed for central nervous system, colorectal, and liver and biliary tract cancers, categorized by sex. The analysis uses the number of diagnosed patients within each cancer category by sex as the denominator. (B) This subsection offers an overview of the epidemiology of these cancer types, delineating the distribution and prevalence by sex, thereby offering insights into the sex-specific epidemiological patterns observed in our study.

# A


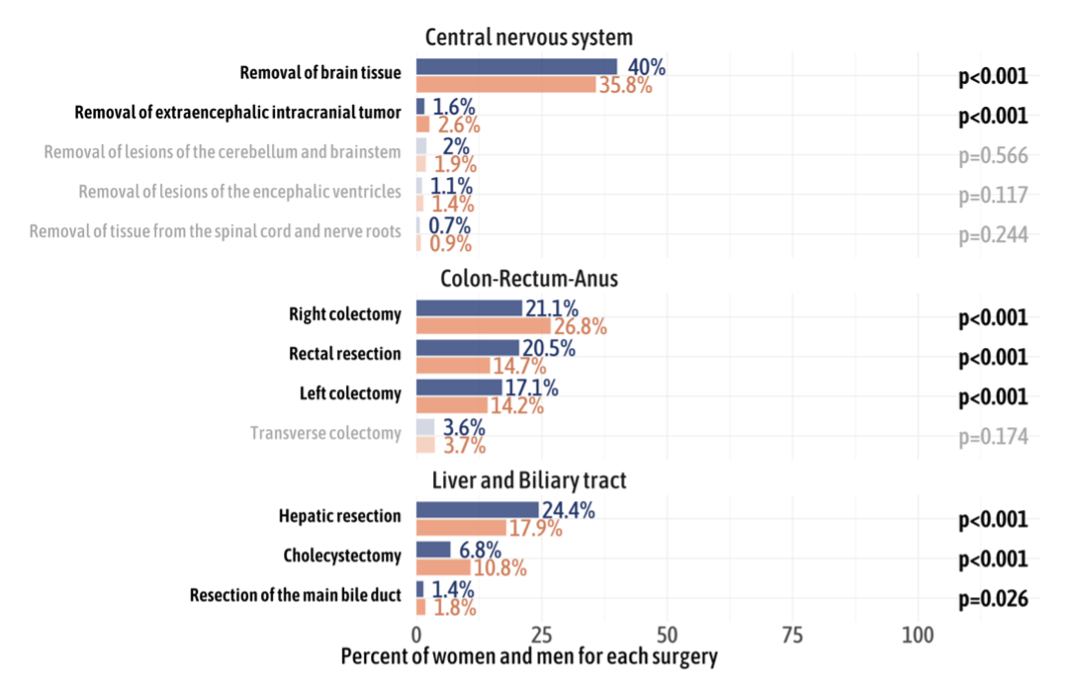


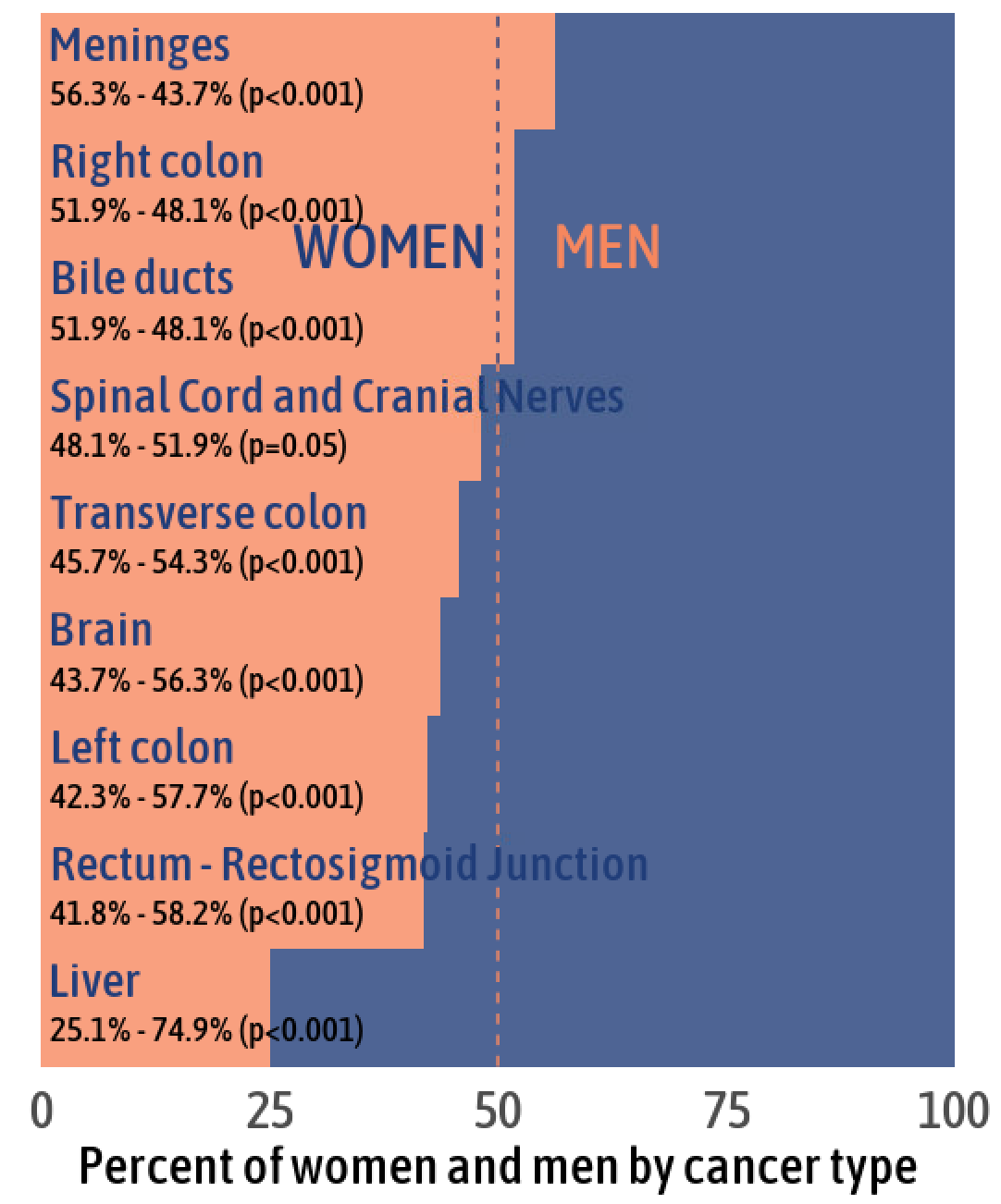
B

# Supplementary Table 1. Findings from the multivariable logistic regression analysis predicting the likelihood of receiving cancer surgery

This model incorporated age, number of comorbidities, and sex as covariates, with the type of cancer included as a random effect. We also checked for interactions between these variables and patient sex. Such an approach allows us to adjust for the specific localization of the cancer, thereby addressing the concern that certain cancer types might disproportionately influence the overall analysis due to their high resection rates.

| **Variable** | **Odds Ratio (95% CI)** | **p value** |
| --- | --- | --- |
| Sex |  |  |
| Men | reference | |
| Women | 1.44 [1.31-1.59] | p<0.001 |
| Age, years | 0.98 [0.98-0.98] | p<0.001 |
| Number of comorbidities | 0.95 [0.95-0.96] | p<0.001 |
| Interaction Sex – Age | 1.00 [0.99-1.00] | p<0.001 |
| Interaction Sex – Number of comorbidities | 0.98 [0.97-0.99] | p<0.001 |

# Random effect: Cancer type

# Supplementary Figure 2. Findings from the multivariable logistic regression analysis predicting 90-day reoperation

After making adjustments for variables such as age, the number of comorbid conditions and the surgical procedure performed, men demonstrated a 21% higher likelihood of undergoing a reoperation within the 90-day postoperative period, as compared to women (OR=1.21). A higher number of comorbid conditions was found to be linked to an increase in the frequency of reoperations within the 90-day postoperative period (OR=1.09).

The surgical procedures associated with the greatest risk of 90-day reoperation included endoscopic destruction and removal of colon lesions, endoscopic destruction of larynx lesion, esophagectomy, extended paryngectomy, and partial pharygectomies and oropharyngectomie.

Similar to the preceding logistic regression, this model was exclusively applied to the top 13 types of cancer.

1. **Focus on sex, age and number of comorbidities**
2. **Complete results**

#

# Supplementary figure 3. Findings from the multivariable logistic regression analysis predicting 90-day mortality

After making adjustments for variables such as age, the number of comorbid conditions and the surgical procedure performed, men demonstrated a 16% higher likelihood of 90-day mortality, as compared to women. An increase of one year in age was associated with a 3% higher likelihood of 90-day mortality (OR = 1.03). Similarly, each additional comorbidity was associated with a 21% increase in the likelihood of 90-day mortality (OR = 1.21).

The surgical procedures associated with the greatest risk of 90-day mortality included removal of lesions of the encephalic ventricles, removal of lesions of the cerebellum and brainstem, total pancreatectomy, pelvectomy and total cystectomy. Surgical procedures with no death are not shown on the figure.

Similar to the preceding logistic regression, this model was exclusively applied to the top 13 types of cancer.

1. **Focus on sex, age and number of comorbidities**

1. **Complete results**

**References**

1 Bannay A, Chaignot C, Blotière P-O, Basson M, Weill A, Ricordeau P, *et al.* The Best Use of the Charlson Comorbidity Index With Electronic Health Care Database to Predict Mortality. *Med Care*. 2016 Feb; **54**: 188–194.

2 Billioti de Gage S, Collin C, Le-Tri T, Pariente A, Bégaud B, Verdoux H, *et al.* Antidepressants and Hepatotoxicity: A Cohort Study among 5 Million Individuals Registered in the French National Health Insurance Database. *CNS Drugs*. 2018; **32**: 673–684.

3 Bouyer B, Rudnichi A, Dray-Spira R, Zureik M, Coste J. Thromboembolic risk after lumbar spine surgery: a cohort study on 325 000 French patients. *J Thromb Haemost*. 2018 Jun 12;

4 Coste J, Blotiere P-O, Miranda S, Mikaeloff Y, Peyre H, Ramus F, *et al.* Risk of early neurodevelopmental disorders associated with in utero exposure to valproate and other antiepileptic drugs: a nationwide cohort study in France. *Sci Rep*. 2020 Dec; **10**: 17362.

5 Giral P, Neumann A, Weill A, Coste J. Cardiovascular effect of discontinuing statins for primary prevention at the age of 75 years: a nationwide population-based cohort study in France. *Eur Heart J*. 2019 Nov 14; **40**: 3516–3525.

6 Jabagi MJ, Vey N, Goncalves A, Tri TL, Zureik M, Dray‐spira R. Risk of secondary hematologic malignancies associated with breast cancer chemotherapy and G-CSF support: A nationwide population-based cohort. *International Journal of Cancer* [Internet]. 2020 Jul 7 [cited 2020 Nov 23]; **n/a**. Available from: https://onlinelibrary.wiley.com/doi/abs/10.1002/ijc.33216

7 Lemaitre M, Kirchgesner J, Rudnichi A, Carrat F, Zureik M, Carbonnel F, *et al.* Association Between Use of Thiopurines or Tumor Necrosis Factor Antagonists Alone or in Combination and Risk of Lymphoma in Patients With Inflammatory Bowel Disease. *JAMA*. 2017 Nov 7; **318**: 1679–1686.

8 Maura G, Bardou M, Billionnet C, Weill A, Drouin J, Neumann A. Oral anticoagulants and risk of acute liver injury in patients with nonvalvular atrial fibrillation: a propensity-weighted nationwide cohort study. *Scientific Reports*. Nature Publishing Group; 2020 Jul 15; **10**: 11624.

9 Sbidian E, Mezzarobba M, Weill A, Coste J, Rudant J. Persistence of treatment with biologics for patients with psoriasis: a real-world analysis of 16 545 biologic-naïve patients from the French National Health Insurance database (SNIIRAM). *Br J Dermatol*. 2019 Jan; **180**: 86–93.

10 Semenzato L, Botton J, Drouin J, Cuenot F, Weill A, Zureik M. Maladies chroniques, états de santé et risque d’hospitalisation et de décès hospitalier pour COVID-19 lors de la première vague de l’épidémie en France: Étude de cohorte de 66 millions de personnes. 2021 Feb 21; 41.

11 World Health Organization. International Classification of Diseases (ICD). Available from: https://www.who.int/standards/classifications/classification-of-diseases
